# Reply to: Altered effort and deconditioning are not valid explanations of myalgic encephalomyelitis/chronic fatigue syndrome

**DOI:** 10.1038/s41467-025-64539-z

**Published:** 2025-10-17

**Authors:** Brian Walitt, Lisa Chin, Bart Drinkard, Samuel Lamunion, Robert Brychta, Kong Chen, Barbara Stussman, Andrew Mammen, Iago Pinal Fernandez, Joseph Snow, Nicholas Madian, Mark Hallett, Avindra Nath

**Affiliations:** 1 National Institute of Neurological Disorders and Stroke, Bethesda, MD USA; 2 Clinical Center, Bethesda, MD USA; 3 National Institute of Diabetes, Digestive and Kidney Diseases, Bethesda, MD USA; 4 National Center for Complimentary and Integrative Health, Bethesda, MD USA; 5 National Institute of Arthritis and Musculoskeletal and Skin Diseases, Bethesda, MD USA; 6 National Institute of Mental Health, Bethesda, MD USA

**Keywords:** Diseases of the nervous system, Physiology, Immunology, Biomarkers

**replying to** T. Davenport et al. *Nature Communications* 10.1038/s41467-025-64538-0 (2025)

Thank you for your interest in our manuscript^1^, and for your comment that our study provides valuable insights into the neurological, immunological, and energy metabolism of myalgic encephalomyelitis/chronic fatigue syndrome (ME/CFS). That alone is a major advancement in the field since based on our findings, several groups have initiated discussions about the design of clinical trials and some studies are underway. We appreciate the opportunity to address your other comments.

The commentary of our study addresses our approach to characterizing and measuring post-exertional malaise (PEM) with cardiopulmonary exercise testing (CPET), our observations of deconditioning and effort preference differences in Post Infection-Myalgic Encephalomyelitis/Chronic Fatigue Syndrome (PI-ME/CFS), and how best to represent and interpret the entirety of our results and the sample size and generalizability of the PI-ME/CFS participants studied.

## Post-exertional malaise and one day versus two day CPET

We agree that PEM is a central concern in the study of ME/CFS. Unfortunately, there is no biomarker for PEM and one is greatly needed in the field. We selected an experimental approach to balance patient safety, the ability to reliably induce PEM, and the ability to collect and analyze samples to study the molecular basis of PEM. To design this portion of the study, we undertook a series of focus groups to develop a new qualitative interview tool to characterize PEM and to understand recovery trajectories. These focus groups suggested that a single CPET is sufficient to reliably induce PEM and that recoveries could be long, with one participant stating she never fully recovered from a 2-day CPET^2^. Reducing stressful exposures in the experimental design was strongly suggested to us in these focus groups. This is particularly important since there are no controlled studies or published guidelines on ‘mitigating the effects of PEM’ after CPET; a review of the website of several of the Commentary authors (Frequently Asked Questions | Workwell Foundation) suggests that these interventions include intravenous saline, bathing in Epsom salts, and diaphragmatic breathing. As the purpose of the experiment was to induce and observe the natural trajectory of PEM, any method to ‘mitigate the effects of PEM’ would defeat and interfere with a major component of the study design.

We were aware that a second CPET day has been used to describe a metabolic abnormality^3^ that includes a continued decline in oxygen consumption, work rate, and heart rate that are considered distinct from physical deconditioning in ME/CFS. While interesting, these measures were not essential for determining case validity or measuring PEM. We decided on a single CPET design that could reliably induce PEM so that its evolution could be serially followed over time with symptom reports, cellular and biochemical sampling, and bioenergetic measurements. We developed a new qualitative interview tool to measure PEM, confirmed successful inducement of PEM in all of the PI-ME/CFS participants^4^, and demonstrated decreases in cardiorespiratory capacity, a lower anaerobic threshold, as well as chronotropic incompetence (Walitt et al. Figure 5 g)^1^ that are consistent with published findings^5^. Replication of the findings speaks to both the large effect size of these phenomena and the validity of our PI-ME/CFS cohort.

We observed that induction of PEM did not alter total body or immune cell bioenergetics over 72 h^1^. We are currently analyzing blood and cerebrospinal fluid samples collected over the same time frame to explore PEM at the level of gene expression, cytokines, and metabolomics. This approach will inform the potential for determining a biological signature of PEM. The use of a single exercise stress makes this analysis straightforward. A second CPET would vastly complicate the interpretation of these results.

## Deconditioning

A major objection of the Commentary authors is that we did not include a group of “deconditioned controls” for comparisons, for which there is no standard. While prior studies assume self-reported activity is sufficient for such a determination^6^, it is not scientifically rigorous. A two-day CPET design with “deconditioned controls” is not the only way nor the best way to measure the impact of physical deconditioning in ME/CFS. The results of a two-day CPET can only be interpreted relative to prior measurements within the same individual. Repeated testing introduces unwanted variability in both the measure and the measured. Quantifiable tissue-based methods for measuring physical deconditioning are preferable to physiological measures, such as two-day CPET.

Contrary to the Commentary authors interpretation of our findings, we did not describe the cardiopulmonary consequences of ME/CFS and physical deconditioning as mutually exclusive. It is important to characterize the individual contributions of both abnormal oxidative metabolism and deconditioning to the phenotype. Using pathologic analysis of the vastus lateralis muscle, we quantified deconditioning as a ratio between the diameter of type II and type I skeletal muscle fibers (Walitt et al. Supplementary Fig. S3D)^1^. These results demonstrate that deconditioning is not required to have PI-ME/CFS. However, this discrete measurement enabled us to explore the impact of deconditioning using correlations (Walitt, et al. Supplemental Fig. 8H–K)^1^. These results show that only a portion of the cardiopulmonary consequences of PI-ME/CFS is associated with muscular deconditioning. Further evidence of muscle deconditioning was noted in network analysis of RNA sequencing data (Walitt et al. Fig. S18)^1^. These data suggest that some skeletal muscle deconditioning over time is a consequence but not the cause of the disorder.

**Fig. 1:**
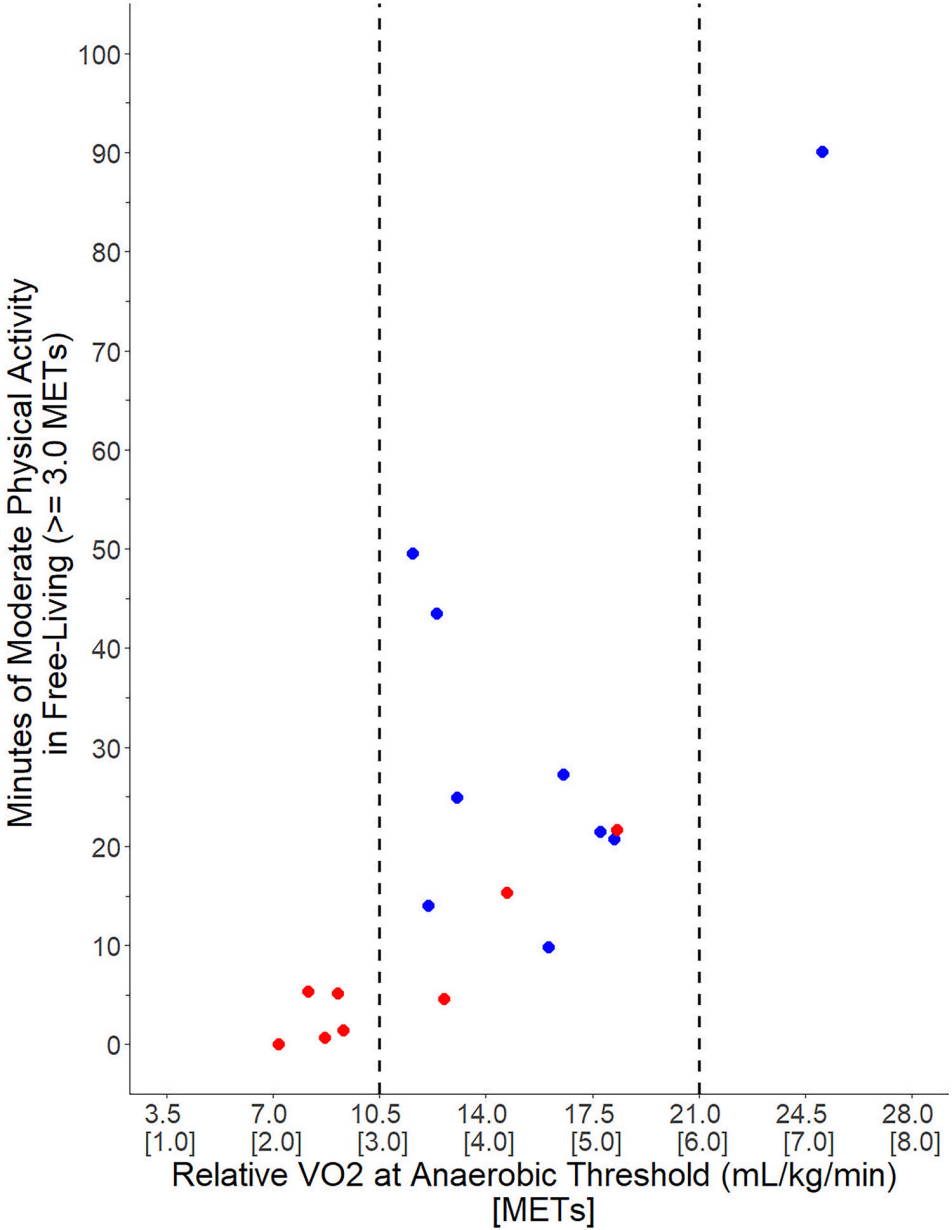
Plot of minutes per day of moderate physical activity (>3.0 metabolic equivalent of task units (METs) and relative VO2 at the anaerobic threshold (mL/kg/min) measured during a cardiopulmonary exercise test (CPET) of PI-ME/CFS participants (red) and healthy volunteers (blue). Values in [brackets] are METs. The dashed lines indicate the lower range [3.0 METs] and upper range [6.0 METs] of the metabolic cost of moderate physical activity. METs data are derived from actigraphy measurements and VO2 data are derived from CPET performed as reported in Walitt, et al.^1^.

Above, we note that the less efficient oxidative respiration measured during CPET and muscular deconditioning are both relevant mechanisms that impact activity in PI-ME/CFS. However, it appeared that a third phenomenon was affecting activity measurements. Across multiple measurements, we noted that impaired physical performance occurs prior to a level of muscular work that taxes oxidative metabolism. This phenomenon can be observed in our CPET and free-living actigraphy data (Welcome - mapMECFS). In Fig. 1, we plot each participant’s daily minutes of moderate activity against their VO_2_ at the anaerobic threshold. Five PI-ME/CFS participants had anaerobic thresholds below 3 metabolic equivalent of task units (METs), the lower limit of moderate intensity for healthy individuals^7^. Of these, two attempted less than one minute of moderate activity a day, suggestive of activity avoidance. Conversely, three participants had anaerobic thresholds above 3 METs. These participants had lower moderate activity counts compared to healthy volunteers with similar anaerobic thresholds.

## Effort preference

Given the stigma faced by patients with ME/CFS we agree that further explanation of the term “Effort Preference” is necessary since the use of this term can be easily misinterpreted and perpetuate stigma. Realizing these issues, we addressed these concerns immediately following the publication of our manuscript, including a frequently asked questions website and a public seminar (https://videocast.nih.gov/watch=54675).

Effort preference is a term that encapsulates the neuroeconomic mechanisms comparing reward and effort cost, as well as the felt experience of those rewards and costs^8–10^. Effort preference should not be interpreted in isolation but rather is part and parcel and the result of numerous other immune, metabolic, neurologic, and autonomic dysfunctions described in these same individuals. Our approach to interpretation was guided by patterns of consilience across all measurements taken. We purposefully avoided making conclusions that were supported only by a single finding.

As oxidative metabolism and deconditioning together did not fully explain the reductions in activity we observed, we further investigated altered physical performance in PI-ME/CFS using grip strength testing. Maximum grip strength was equal between the groups while the ability to sustain grip force was markedly reduced. If deconditioning were the cause of the performance difference, we would have expected a difference in maximum strength. To better understand the contributions of altered oxidative metabolism and effort preferences to this difference in sustained grip, we performed a series of repetitive grip tasks using functional magnetic resonance imaging (fMRI) and transcutaneous magnetic stimulation (TMS) paradigms. These experiments were designed to induce muscle and motor cortical fatigue while measuring biomarkers of muscle respiration and brain activity in a setting with minimal encouragement or reward offered. When performing the repetitive grip task, PI-ME/CFS participants gripped with less sustained force and stopped performing the task much earlier than HVs (Walitt et al. Fig. 4 A)^1^. In the PI-ME/CFS participants, performance ceased without measurable muscle fatigue. The Dimitrov Index, a dynamic measure of muscle fatigability that is a marker of the consequences of oxidative metabolism, increased as expected in healthy volunteers when they began to show signs of performance impairment. The opposite was seen in PI-ME/CFS participants with a decrease in Dimitrov Index (Walitt et al. Fig. 4B)^1^. In PI-ME/CFS, grip performance failure was not due to less efficient oxidative respiration. If this were the case, the Dimitrov Index would have increased at a higher rate than that of the healthy volunteers. Altered oxidative respiration in the muscle was not the cause of the altered ability to sustain motor performance.

The difference in sustaining grip force was also not a consequence of motor cortical fatigue. Similar to our fatigability results in muscle, a divergent pattern of activation was observed in the motor cortex (Walitt et al. Fig. 4 C)^1^. The HVs continued to increasingly activate their motor system while the PI-ME/CFS decreased their motor activation as the participant’s grip approached failure. Diminished PI-ME/CFS group performance was not related to motor cortex fatigue. Rather, it was related to a failure to activate the task-related neuronal circuits. This failure was related to altered functioning of the temporoparietal junction, an integration brain region with roles in movement and our sense of will^11–13^. We were unable to demonstrate fatigue but were able to demonstrate the neuronal correlates of an unfavorable effort preference.

Altered effort preference was also observed using the Effort-Expenditure for Rewards Task. This task uses the chance of small monetary awards as encouragement, in contradistinction to the strong verbal encouragement during CPET and the minimal encouragement during the grip strength experiments. We observed an Odds Ratio of 1.65, *p* = 0.04 for the probability of selecting a hard task rather than an easy one, a medium-sized difference in effort preference. We observed no differences in the rate of fatigue between the groups during the task (Walitt et al. Fig. S5)^1^, or during the three-hour neuropsychiatric battery it was part of (Walitt et al. Fig. S10)^1^, suggesting that PEM did not impact these results.

In the commentary, the Commentators “challenge [Walitt, et al.]’s conclusions by suggesting the key symptom, exertion intolerance, along with the patient’s strategy to avoid the debilitating PEM, are the most likely explanations for the patient’s reduced activity.” We agree and posit that the strategy described by the Commentators is an effort preference. As explained above this effort preference is not an overt ‘voluntary’ strategy nor it is an intentional basis for exerting less energy. As stated in our paper, “Conscious and unconscious behavioral alterations to pace and avoid discomfort may underlie the differential performance observed”. It seems that, in essence, the Commentators agree with us.

## Cohort size and PI-ME/CFS severity

We agree that the sample size of our cohort was not sufficiently powered to address each of the numerous tests done on this population. However, the cohort was very well characterized, which allowed us to make several seminal discoveries with large effect size that would not have been possible otherwise. We also agree that our cohort is not fully representative of the whole range of the ME/CFS population, as that was not the intent of this project. Our program was an exploratory deep phenotyping effort to characterize PI-ME/CFS, where each participant was in good health before contracting an infection that led to the onset of ME/CFS. To ensure case validity, we employed a rigorous screening process with the goal of recruiting an unbiased PI-ME/CFS cohort. Confounding factors from medical and psychiatric comorbidity were removed to the best of our ability. Temporal attribution of ME/CFS was directly related to an infection with medical record documentation. Further, the cases themselves were so clear to be PI-ME/CFS after an exhaustive in-person evaluation that a panel of clinical experts could unanimously agree to their validity. While the cohort size is small, we assert that it is the purest representation of PI-ME/CFS studied to date.

With regards to the severity of the illness of the individuals in our cohort, nearly all participants had severe disease as per patient-reported outcome measurements^14^, in part because substantial symptom severity was an inclusion criterion (Walitt et al. Supplementary Data 1)^1^. In general, participants had ME/CFS symptoms two standard deviations more severe than healthy volunteers (Walitt et al. Supplementary Data 7)^1^. Further, the individual descriptions of PEM severity were reviewed by our expert panel as part of the adjudication process. We assert that each participant included in our cohort represented a moderate to severe case of PI-ME/CFS.

In conclusion, our data shows that fatigue, altered aerobic respiration, and muscle deconditioning are not the features that best define the moment-to-moment motor behavior of PI-ME/CFS. This seems to be a crucially important observation in a disorder currently labeled ‘Chronic Fatigue Syndrome’. A better understanding of the biology of effort preference, rather than the biology of fatigue, may be the science that best serves those with PI-ME/CFS.
